# Assessment of posture and joint movements of the upper limbs of patients after mastectomy and lymphadenectomy

**DOI:** 10.1590/S1679-45082013000400004

**Published:** 2013

**Authors:** Cinira Assad Simão Haddad, Marcelo Saad, Maria del Carmen Janeiro Perez, Fausto Miranda

**Affiliations:** 1 Universidade Federal de São Paulo, São Paulo, SP, Brazil.; 2 Hospital Israelita Albert Einstein, São Paulo, SP, Brazil.

**Keywords:** Posture, Lymphedema/etiology, Mastectomy/adverse effects, Upper limb, Articular range of motion /pathophysiology

## Abstract

**Objective:**

To evaluate alterations in posture and range of motion of the upper limbs in women after mastectomy and lymphadenectomy, submitted to radiotherapy as adjuvant treatment.

**Methods:**

Two groups were evaluated: 16 post-mastectomy women with lymphedema of the upper limb and 14 post-mastectomy women without lymphedema. Patients were submitted to analysis made by software, one for posture and the other to measure ranges of movement of the shoulder, elbow, and wrists. The results obtained were compared between the right and left sides, and operated and non-operated sides, and then were submitted to statistical tests.

**Results:**

Both groups presented with anteriorization of the trunk. The women with lymphedema had head rotation to the right, protrusion of the left shoulder, and trunk inclination angle smaller on the operated side, besides bilateral elevation of the scapula when compared to the group with no lymphedema. Changes in range of motion were also smaller on the operated side in terms of flexion, abduction, and external rotation of the shoulder for all women, and for those with lymphedema, elbow extension and wrist flexion had a smaller range of motion.

**Conclusion:**

Women submitted to mastectomy presented with asymmetries and modifications in posture, and lymphedema seemed to worsen this condition. Additionally, they had deficits in range of motion in the shoulders on the operated side. Women with lymphedema also showed deficits in the elbows and wrist.

## RESUMO

**Objetivo:**

Avaliar as alterações posturais e de amplitudes de movimento de membro superior nas mulheres pós-mastectomia e linfadenectomia que se submeteram à radioterapia como tratamento adjuvante.

**Métodos:**

Foram avaliados dois grupos: 16 mulheres pós-mastectomia com linfedema de membro superior e 14 mulheres pós-mastectomia sem linfedema. As pacientes foram submetidas à avaliação feita por programas computadorizados, um para postura e outro para medir as amplitudes de movimento de ombro, cotovelo e punho. Os resultados obtidos foram comparados entre lado direito e esquerdo, e operado e não operado, sendo submetidos a testes estatísticos.

**Resultados:**

Ambos os grupos apresentaram anteriorização de tronco. As mulheres com linfedema mostraram rotação de cabeça à direita, protusão de ombro do lado esquerdo e medidas do ângulo de talhe menores do lado operado, além de elevação da escápula bilateralmente, quando comparadas ao grupo sem linfedema. As alterações de amplitude de movimento também foram menores do lado operado na flexão, abdução e rotação externa de ombro para todas as mulheres e, para aquelas que tinham linfedema, a extensão de cotovelo e a flexão de punho tiveram menor amplitude de movimento.

**Conclusão:**

Mulheres que foram submetidas à mastectomia apresentaram assimetrias e alterações de postura, e o linfedema parece agravar essa condição. Além disso, apresentaram déficits de amplitude de movimento em ombros, do lado operado. Mulheres com linfedema exibiram também déficits em cotovelo e punho.

## INTRODUCTION

Breast cancer is one of the most frequent neoplasms among women. Currently, early diagnosis and technological advances allow treatment and survival of patients and consequently there is a concern about postoperative complications^(1)^. In this way, some studies have been made to demonstrate these complications, as well as their influence on the daily of lives of women after treatment^(2–10)^.

After mastectomy, women have more problems than those with a local incision, as well as patients who undergo radiation therapy as compared to those who do not have this treatment^(2)^. Morbidity of the upper limb is high due to axillary lymph node dissection, increasing the chances of lymphedema and decreased sensation of the axilla^(3)^. The association between mastectomy and radiation therapy leads to significant reduction of the arches of movement for all movements of the shoulder^(4)^.

In mastectomies, body posture will be affected, especially if the patient has large and heavy breasts. There is muscle contraction of the cervical and scapular regions triggered by emotional stress, associated with musculoaponeurotic retraction of the muscle masses involved, cause by postoperative scars or by post-radiation therapy fibrosis. Women feel difficult to perform some activities of daily life with the affected upper limb and perceive some posture disorders^(5,11)^.

Removal of axillary lymph nodes is the primary risk factor, and when it is followed by postoperative radiation therapy, it significantly increases the risks of lymphedema^(12)^. A chronic disease, lymphedema is characterized by accumulation of interstitial fluid and tissue alterations resulting from insufficient lymph drainage^(13)^.

Postoperative lymphedema after breast cancer is a secondary lymphedema, that alters lymph drainage of the breast, thoracic quadrants, and upper limbs^(11)^. Its signs and symptoms include increased weight of the limb; paresthesia of the hand; stiffness of fingers; reduced range of motion of shoulder, elbow, and wrist; increased incidence of infections; posture deformities; limited function; and psychological and emotional problems. In the post-mastectomy phase, these symptoms are worsened by pain at the incision site, in the posterior cervical area, shoulder girdle, and in scar adhesions; muscle weakness of the upper limb and shoulder girdle; postural defects, such as kyphosis and scoliosis due to poor habits, generating trunk asymmetry and restricted mobility of the shoulder^(14)^.

## OBJECTIVE

To evaluate the alterations in posture and range of motion of the upper limb in post-mastectomy women who underwent radiation therapy as adjuvant treatment, in addition to verifying if lymphedema worsens this conditions.

## METHODS

This was a prospective, quantitative, study carried out at the *Escola Paulista de Medicina da Universidade Federal de São Paulo* (UNIFESP), between February and December 2007, with post-mastectomy patients. The study was approved by the Research Ethics Committee of the UNIFESP, under number 1415/05. All patients involved were aware of the objectives of the study and signed the Informed Consent Form.

Posture and range of motion evaluations were made on these patients by means of two software, both noninvasive. One of the programs assessed posture, measuring and quantifying posture deviations; the other evaluated measurements of range of motion, performing an angular analysis of joints, examining joint mobility, and measuring, in degrees, the range of each movement. Thirty women were evaluated and divided into two groups: the group with mastectomy and lymphedema (Group ML) had 16 patients, and the group with mastectomy and no lymphedema (Group M) had 14 patients.

The inclusion criteria were patients submitted to total, radical, or modified radical mastectomy, with axillary lymph node dissection, at least 6 months before and up to 5 years of the evaluation date, aged between 18 and 70 years, who underwent radiation therapy. The exclusion criteria were quadrantectomies, sentinel node biopsy without axillary dissection, peripheral nerve damage, neurologic diseases, cognition deficits, history of significant orthopedic problems, breast reconstruction, patients who did not submit to radiation therapy, those engaged in activities that could originate posture asymmetries or who had undergone any type of treatment for posture correction.

In order to define the presence or not of lymphedema, a minimal difference of 2cm between the right and left limb circumference of each patient^(15)^in at least two measurements was considered. Six measurements (perimetry) were made in each upper limb in all patients, namely: dorsum of the hand, proximal phalanx of the third finger, and starting with the 3rd finger, 20cm, 30cm, 40cm, and 50cm from distal to proximal, ascending the upper limb.

Assessment was made individually. Demarcation of previously established evaluation points, generally at bony prominences, was made with circular stickers. For posture evaluation, the following were observed: acromion; antero-superior iliac spine; lateral malleolus; inferior angle of the scapula; postero-superior iliac spine, and glabella. For range of motion, these were evaluated: superficial projection of the center of the glenohumeral joint laterally; greater and lesser tubercles of the humerus; center of the olecranon; ulnar styloid process; acromion; superficial projection of the center of the elbow joint; and center between radium and ulna, distally.

Patients were photographed and the images were captured by a digital camera (Sony, Cyber-Shot, 4.1 mega pixels) always supported on a tripod (Lightweight Tripod). This tripod was used so there would be no lateral, diagonal, or vertical deviations of the camera. Distance from the camera to the patient was not important since the software used for assessments allows adjustment of a scale for each image, according to an object with known length placed on the patient's body. This object is a 10-cm-long white ruler, provided by the same software company.

With the patient in orthostatic position, posture examination was carried out with capture of six images: ventral, posterior, left and right profile surfaces, and anterior flexion of the trunk, with anterior and lateral views. The posture evaluation software (*Posturograma Clínico, Fisiometer^®^*, version 2.8) emitted reports with photos and graphic evaluation, so that these data were evaluated quantitatively, since the software makes exact measurements between the points marked. Posture alterations were compared between the two groups ML and M, taking into consideration the right and left sides, operated and non-operated, comparing one side to the other, as well as the relation between the groups.

Assessment of joint range of motion actively evaluated movements of the shoulder girdle and upper limbs, with protocols already defined by the software itself. In this way, the position of the patient for evaluation was sitting, with a 90° flexion of the hip, or standing up, on the frontal and lateral planes. Image capture was always made so as to allow visualization of the joint range of motion to measure the angles: shoulder: flexion, extension, adduction, abduction, internal and external rotation; elbow: flexion, extension, pronation, and supination; and wrist: flexion, extension, ulnar and radial deviation. The software to evaluate articular range of motion (*Fotogoniômetro, Fisiometer^®^*) issued reports with photo shots and the patient's graphic evaluation; data were evaluated quantitatively, in degrees.

Information on alterations in range of motion were compared between the ipsilateral and contralateral limbs relative to each patient's operation, comparing the difference between the groups, since the limb contralateral to the operation of each patient served as measurement to establish a range of normality. Additionally, the dominant and non-dominant sides were compared, which coincided with the right and left sides, respectively, for all patients in Groups ML and M. In this way, the groups were divided when the dominant side was the one operated on and vice-versa.

For analysis of the results, the following tests were applied: Wilcoxon (Siegel) or paired *t* test (Zar) to compare measurements made between the right and left sides, operated on and not operated on; independent t test (Zar) or Mann-Whitney's test (Siegel) to compare the measurements of independent groups; Kruskal-Wallis (Siegel) variance analysis or analysis of variance for independent groups (Zar) to compare the two groups for the variables studied. The level of rejection of the null hypothesis was set at 0.05 or 5%. A descriptive analysis was made to demonstrate the percentage of modifications in each group.

## RESULTS

There was no difference between the groups as to age (ML=58.9 and M=59.7), body mass index (ML=27 and M=26.4), and time since operation (ML=50 months and M=56 months).

The types of procedures involved in the study were: in the ML Group, seven patients (47.75%) underwent radical mastectomy, and nine (56.25%) were submitted to modified radical mastectomy; in Group M, six patients (42.85%) were submitted to radical mastectomy, and eight (57.15%) underwent modified radical mastectomy – with no difference between the groups as to the type of surgery. Table 1 shows the comparison between the right and left sides of the patients. Measurements from the posterior apex (PA) until the plum line (PL) (PA-PL) were statistically significantly greater on the right side compared to the left in both groups. In Group ML, measurements from the PL to the ear pinna (EP) (PL-EP) and from the PL up to the greater tubercle of the humerus (Tu) (PL-Tu) also showed significant differences, in which the left side was greater for both measurements. In Group M these two measurements, PL-EP and PL-Tu, displayed no statistically significant differences, but the left side was also greater in both measurements.

**Table 1:** Comparison between the right (R) and left (L) sides, according to posture evaluations of post-mastectomy patients with lymphedema (ML) and without lymphedema (M). Results from the Wilcoxon test, paired t test or independent t test

Evaluated items	Means	Group ML			Group M		
		R	L	Total	R	L	Total
PA-LS	Mean	5.21	4.86	p=0.163	5.01	4.78	p=0.382
	Median	5.06	4.85	NS	4.89	4.52	NS
PA-PL	Mean	11.8	7.77	p=0.001	10.27	7.40	p=0.019
	Median	11.45	7.12	R>L	9.67	7.13	R>L
PL-EP	Mean	1.17	6.03	p=0.02	3.58	6.44	p=0.056
	Median	0.97	6.43	R<L	3.03	7.98	NS
P L -Tu	Mean	3.46	6.78	p=0.044	4.68	7.21	p=0.074
	Median	2.45	7.77	R<L	4.31	8.75	NS
Scapula height	Mean	1.08	1.08	p=0.544 NS	1.01	1.01	p=0.054 NS
Trunk inclination angle	Mean	1.55	1.39	p=0.578 NS	1.65	1.45	p=0.320 NS

NS: not significant; PA: posterior apex; LS: lumbar spine; PL: plum line; EP: ear pinna; Tu: greater tubercle of the humerus.

Comparing the items evaluated in both groups as to right and left sides (Table 2), the height of the scapula was statistically significant, and it was higher in Group ML, both on the right and the left sides. Other measurements evaluated that were significant on the right side were PL-EP, which was greater in Group M. The measurement of PA-PL, despite not numerically significant (p=0.051), shows that Group ML had this greater measurement than Group M, only on the right side. Although also not statistically significant between the groups, the measurements of shoulder-floor, shoulder-pelvis, and pelvis-floor also demonstrated a difference, which was greater for Group ML relative to M, both on the right and left sides. The trunk inclination angle also was not a statistically significant measurement, but the right side was smaller than the left in all groups.

**Table 2:** Comparison between the groups of post-mastectomy patients with lymphedema (ML) and without lymphedema (M) relative to right (R) and left (L) sides, according to posture assessments. Result of Kruskal-Wallis (Siegel) test or t test for independent groups (Zar)

Evaluated items		R			L		
		ML	M	Total	ML	M	Total
PA-LS	Mean	5.21	5.01	p=0.967 NS	4.86	4.78	p=0. 739 NS
	Median	5.06	4.89		4.85	4.52	
PA-PL	Mean	11.80	10.27	p=0.051 NS	7.77	7.40	p=0.547 NS
	Median	11.45	9.67		7.12	7.13	
PL-EP	Mean	1.17	3.58	p=0.038 M>ML	6.03	6.44	p=0.48 NS
	Median	0.97	3.03		6.43	7.98	
P L-Tu	Mean	3.46	4.68	p=0.151 NS	6.78	7.21	p=0.36 NS
	Median	2.45	4.31		7.77	8.75	
Scapula height	Mean	1.08	1.01	p=0.038 ML>M	1.08	1.01	p=0.032 ML>M
Trunk inclination angle	Mean	3.46	4.68	p=0.694 NS	6.78	7.21	p=0.847 NS

PA: posterior apex; LS: lumbar spine; NS: not significant; PL: plum line; EP: ear pinna; Tu: greater tubercle of the humerus.

When comparing the operated and contralateral sides, only one item evaluated showed a statistically significant difference between them (p=0.001): in Group ML, the trunk inclination angle was smaller on the operated side than on its contralateral. In Group M, no item had any significant difference between the operated and non-operated sides.

In the comparison between groups (ML and M), for the side operated and not operated, the only result with significant differences was the height of the scapula, with measurements greater in Group ML, both for the operated and non-operated sides (Table 3).

**Table 3:** Comparison between post-mastectomy patients with lymphedema (ML) and without lymphedema (M), relative to the side operated and not operated on according to the items of posture evaluated. Results of the Mann-Whitney test (Siegel) and independent t test

Evaluated Items	Means	Operated			Non-operated		
		ML	M	Total	ML	M	Total
PA-LS	Mean	15.75	15.21	p=0.886 NS	15.88	15.07	p=0.822 NS
PA-PL	Mean	16.0	14.93	p=0.759 NS	17.41	13.32	p=0.208 NS
PL-EP	Mean	15.25	15.79	p=0.886 NS	12.97	18.39	p=0.093 NS
PL -Tu	Mean	15.22	15.82	p=0.854 NS	13.44	17.86	p=0.179 NS
Scapula height	Mean	1.08	1.01	p=0.033 ML>M	1.08	1.01	p=0.036 ML>M
Trunk inclination angle	Mean	1.08	1.56	p=0.143 NS	1.86	1.54	p=0.160 NS

PA: posterior apex; LS: lumbar spine; NS: not significant; PL: plum line; EP: ear pinna; Tu: greater tubercle of the humerus.

In glabella displacement, in Groups ML and M, 100% of the women had deviation to the side.

When observing height of the shoulder relative to the floor, in both groups the non-operated side was higher than that of the contralateral side with greater frequency −50% in Group ML and 43% in M. Five (36%) patients in Group M showed no difference between these measurements bilaterally, whereas in Group ML, only two (12.5%) were symmetrical for this measurement.

The measurement of the height of the scapula showed the operated side always higher in both groups (37.5% for ML and 28.5% for M).

Measurement of the trunk inclination angle showed, in Group ML, only one (6.3%) patient with equal measurements bilaterally. Three patients (19%) were larger on the operated side in contrast with 12 (75%) that were not. In Group M, five (35.5%) patients had the same angle distance measurements bilaterally, three (21.5%) presented with this measurement greater on the operated on side and six (43%) had measu rement greater on the non-operated on side. There was no difference between the right and left sides.

As to the distance between the scapula and the spine, which can show protrusion of the shoulder and/or weakness of dorsal musculature, in Group ML, nine (56.2%) patients had this measurement greater on the operated on side, and in Group M, on the operated side there were eight (57%) patients with greater measurements.

As to the measurement of PA-PL, which may show posterior or anterior dislocation of the body relative to the center of gravity, it was noted that most patients had this measurement greater on the right side, which shows the influence of the dominant side in determining the rotation of the trunk. Only one patient from each group showed this measurement within range of normality (6 to 8cm) for the right side; the rest were greater.

The PL-EP measurements from the head to the PL were greater than what is considered normal, *e.g.*, an anteriorized position of the head. In Group ML, 10 (62.5%) patients had measurements greater than normal for the right side and 14 (87.5%) were greater for the left side; 13 (81.2%) patients showed this measurement greater for the side operated on, and 11 (68.8%) for the side not operated on. In Group M, 13 (93%) patients had this measurement greater on the left side and 13 (93%) on the right side, and these same percentages appeared on the operated and non-operated sides.

The right side was dominant in 100% of the patients. In Group ML, 56% (9) of the patients had surgery on their left side, against 44% (7) with surgery on the right. In Group M, 57% (8) of the patients had surgery on the left side against 43% (6) on the right.

Table 4 shows the summary of statistically significant movements, considering dominance of the patients and the side of the operation. For Groups ML and M, the movements marked were smaller than their contralateral sides.

**Table 4:** Summary of movements with a statistically significant difference relative to the contralateral side, taking into consideration dominance and the operated side. Wilcoxon test, with Z>1.96 and level of significance 5%

	Dominance				Surgery	
Movements	Group ML (n=16)		Group M (n=14)		Group ML (n=16)	Group M (n=14)
	Dominant operated < contralateral (n=7)	Non-dominant operated < contralateral (n=9)	Dominant operated > contralateral (n=6)	Non-dominant operated < contralateral (n=8)	Side operated < contralateral	Side operated < contralateral
Flexion of the shoulder	Z=2.37 p=0.009	–	–	–	Z=2.71 p=0.003	Z=2.17 p=0.015
Extension	–	–	–	–	–	–
Adduction	–	–	–	–	–	–
Abduction	–	Z=2.31 p=0.01	–	–	Z=2.33 p=0.01	Z=1.98 p=0.005
Internal rotation	Z=2.37 p=0.009	–	–	–	–	–
External rotation	–	Z=2.67 p=0.004	–	–	Z=2.90 p=0.002	Z=1.98 p=0.005
Flexion of the elbow	–	–	–	–	–	–
Extension of the elbow	–	–	–	–	Z=2.41 p=0.008	–
Pronation	–	–	–	–	–	–
Supination	–	–	–	–	–	–
Flexion of the wrist	–	Z=2.43 p=0.008	Z=2.20 p=0.014	–	–	–
Extension of the wrist	Z=2.37 p=0.009	–	–	–	–	–
Radial deviation	–	–	–	–	–	–
Ulnar deviation	Z=2.20 p=0.014	–	–	–	–	–

ML: post-mastectomy patients with lymphedema; M: post-mastectomy patients without lymphedema.

When the dominant side underwent surgery in Group ML (n=7), the statistically significant movements - with reduced range of motion as compared to the contralateral side - were shoulder flexion, internal rotation of the shoulder, extension of the wrist, and ulnar deviation of the wrist. For this same comparison, in Group M (n=6), there was only one movement with a statistically significant difference, flexion of the wrist, and for this movement, the affected side was greater than its contralateral side.

In comparing the operated non-dominant side and its contralateral side, there was a statistically significant difference in abduction of the shoulder, external rotation of the shoulder, and wrist flexion, in which the operated side was always smaller than the other, all in Group ML (n=9), whereas in Group M, there was no difference for this type of comparison.

When only the operated side and its contralateral are compared, without considering dominance of the patients, the statistically significant differences were observed in shoulder flexion, shoulder abduction, external rotation of the shoulder for Groups M and ML – all with the side affected by the operation having smaller measurements relative to the contralateral. Group ML also presented with a statistically significant reduction for the extension of the elbow (p=0.008).

## DISCUSSION

The present study showed a few alterations of posture and of upper limbs common in women who have undergone surgery for the treatment of breast cancer. Problems such as lymphedema, pain, parenthesia, decreased muscle strength, and reduced range of motion of the member affected are frequently observed and reported by women who underwent breast surgery^(1)^. The purpose of the study was to use a method that could allow quantifications of deviations and deficits of range, generating measurable physical data for better study results.

Significant complications after mastectomy are alterations in body posture caused by disorders in body posture, as a result of amputation and limitation of motion, besides the painful state of the spine^(6)^. Posture may also suffer alterations due to the psychological aspect, from the sensation of mutilation and fear of pain^(11)^.

Pereira^(16)^ conducted a preliminary study to validate a computerized analysis as a method of assessing posture alterations. In evaluating 50 individuals by means of medical history and physical/clinical examination of posture, and later, using the method of computerized posture analysis, he obtained as a result most of the items evaluated consistent between the two types of evaluation, in the same population studied, and concluded that this is an easily applied appropriate methodology, but it that depends on the management and conscientious interpretation of the results, which should be made by a qualified professional.

By means of photogrammetry, Malicka et al.^(17)^evaluated the postures of women after mastectomy comparing them to healthy women, and observed that 82.3% of women after treatment presented with posture failures, against 35.1% in healthy women.

In this study, posture alterations were also found in patients after mastectomy. In both groups it was confirmed that the PA-PL measurement was greater on the right side than on the left. This measurement demonstrates an anteriorization of the trunk or a dislocation of the center of gravity to the front, evidencing a rotation of the trunk towards the right side, which probably is defined by the influence of dominance in these women. All women in Groups ML and M had their right side as dominant. Bricot^(18)^ says that the rotation of the shoulder girdle is strongly influenced by laterality - for the right-handed person, the right shoulder girdle is more anteriorized. In Groups ML and M, 93.7 and 93%, respectively, had this anteriorization of the trunk predominantly to the right side. As to the side of the surgery, it was noted that most of the patients not operated on had these greater measurements (68.7% in Group ML and 71.5% in Group M).

Rostkowska et al.^(6)^ also observed these data in a study comparing healthy and post-mastectomy women. After this operation, the women had a greater anterior inclination of the trunk than the healthy ones. It was also noted that women with a more recent operation had greater anteriorization of the trunk than those who had undergone surgery much earlier, and this fact was associated with the adoption of a more anteriorized posture, with intention of analgesia and a position of protection.

In this study, we also point out that in Group ML, the measurement PL-EP was statistically smaller on the right side. This shows anteriorization or anterior projection of the head in women with lymphedema relative to the others, and head rotation to the right side, and since the measurements on that side were smaller, there is evidence of a shorted distance of this measurement on that side. The weight of the limb due to lymphedema may make the musculature of the shoulder and shoulder girdle become retracted, tensioning cervical and neck muscles, and making the head incline and rotate. These alterations may be linked to the adoption of this posture due to pain, skin and surgical scar retraction, or psychological reaction^(14)^. It may also be associated with retraction of the cervical muscles, caused by emotional stress, contractures of the trapezium, scalene, and interscapular muscles and musculoaponeurotic retraction of the muscle groups involved – and reduction in elastic properties and fibrosis of the skin due to radiation therapy^(11)^.

Another statistically significant measurement in Group ML was PL-Tu, in which the right side was smaller than the left. Again, these results suggested that in women with lymphedema there was anteriorizationof the trunk or of the upper limb greater than in Group M, influencing protrusion of the shoulder. Camargo and Marx^(11)^ reported that the lack of weight of the breast will make the shoulder on the operated side elevate and gyrate internally, abducing the scapula and causing muscle contracture of the cervical region – and consequently, pain. The greater measurements on the left side suggested a greater anteriorization or internal rotation of the shoulders to the left side, since these were greater than on the left side.

In the comparison between the groups, height of the scapula was greater both on the right and the left side, but only in Group ML. This suggested elevation of the scapula in these women, but it does not differentiate the dominant or contralateral sides. PL-EP measurement also appears as significantly greater in Group M and only on the right side. This fact suggests that in post-mastectomy women with no lymphedema, there was greater rotation of the head to the left side, since the greater measurement on the right side indicated rotation to the opposite side.

Although not statistically significant, but very close to this, PA-PL measurement is also noted as greater in women of the ML Group, on the right side, indicating greater trunk anteriorization in them. Other measurements also not significant, but that show alterations present only in women from Group ML in comparison with Group M, are the shoulder-floor, shoulder-pelvis, and pelvis-floor measurements, a fact that suggested a lateral asymmetry in the trunks of these women, especially related to height of the shoulder to the floor and to the pelvis, indicating lateral inclination of the trunk or scoliosis. The greater height of the scapula also in Group ML confirms this datum. Camargo and Marx^(11)^ reported that pain and tension due to carrying greater arm weight in the presence of lymphedema, may increase the adoption of a posture with more elevated shoulders.

Kisner and Colby^(14)^ reported that, in patients after mastectomy, trunk asymmetries and abnormal alignment of the scapulas may occur as a result of a sudden change in lateral weight, especially in women with voluminous breasts. Rostkowska et al.^(6)^ also detected a difference in heights of scapulas in post-mastectomy women, in which they were greater on the operated side, when compared with healthy women. Also reported was that the presence of lymphedema contributes to intensify disorders in body posture, in agreement with the data of this study.

When comparing the operated side and the non-operated side of each patient, the measurement of the trunk inclination angle proved smaller on the operated side, with statistically significant measurements. The trunk inclination angle is the distance between the lateral surface of the trunk to the upper limb on the same side, in orthostatic position. The data in this study revealed that the trunk of post-mastectomy patients with lymphedema is laterally inclined to the side opposite the surgery, since the smaller the measurement of this distance, the greater the inclination of the trunk towards the opposite side. In this way, the height of the scapula on the operated side also becomes greater, which corroborates the data obtained as to more elevated height of the scapula on the operated side. Additionally, data from measurements of shoulder to pelvis showed that in Group ML, 19% of the women had equal measurements bilaterally for this item, and 56% had the operated side with greater measurements than the contralateral side. In Group M, 14.5% were equal measurements and 50% were greater on the operated side. This demonstrates a greater rate of trunk inclination to the side opposite to mastectomy operation, potentializing elevation of the scapula on this same side, regardless of the presence of lymphedema.

The present study evaluated the ranges of movement of the upper limbs of patients. Taken into consideration was the dominance of the patients in assessing range of motion, since it is known that this may cause interference in range of motion and posture, due to greater use of the dominant side for some activities, potentially generating, in more common movements, increased range of motion^(18,19)^.

When the operated dominant side was analyzed, some movements with range of motion smaller than the contralateral side were noted only in Group ML: shoulder flexion, internal rotation, wrist extension, and ulnar deviation. In this case, it was suggested that the lymphedema, and not only the mastectomy, was affecting these movements.

In Group M, the operated dominant side had greater wrist flexion than the contralateral side, which suggested increased use of the wrist on the dominant side as compensation for the restriction of some shoulder movements on the operated side.

In the analysis of the operated non-dominant side, shoulder abduction and external rotation, and wrist flexion were statistically smaller in Group ML, whereas in Group M, there was no difference in any movement when the non-dominant side was operated on. Again it may be suggested that the lymphedema has more influence on the reduction of these ranges than the operation itself. Smaller wrist flexion may be associated with lesser use in some manual activities, since besides not being dominant, it is the operated side that possibly is avoided for performance of great efforts. The forces of clamping and gripping generally become diminished on the operated side of the women, as a result of lymphedema and secondary stiffness of the fingers^(8,14)^. Post-mastectomy women suffer body image alterations, and fear of experiencing pain and the possibility of disability make them display greater difficulty in carrying out the exercises^(11)^.

There are not many studies that confirm the influence of lymphedema on movement deficits, but clinical practice shows a significant complaint, on the part of the patients, regarding performing certain movements, especially carrying weight or in raising the limb against gravity, since the weight of the limb increases due to accumulation of fluid and proteins^(20)^.

When the dominance of the patients is not taken into consideration, comparing only the operated side with its contralateral, some movements, such as flexion, abduction, and external rotation of the shoulder, appear as statistically smaller, both the in Group ML and in Group M. Even so, the statistical values found in Group ML were lower than in Group M, which suggested that even with the restriction of these movements in both groups, in the lymphedema group, the restriction was greater. Elbow extension was smaller on in Group ML, which suggested that the lymphedema had influence on the total range of motion of this joint. Assunção and Mello^(21)^ had already stated that upper limb post-mastectomy lymphedema causes numerous consequences, such as decreased muscle strength and range of motion of the joints involved.

Camargo and Marx^(11)^ declared that it is abduction and anterior flexion of the shoulder that are limited, as well as external rotation associated with abduction, confirming the results of this study. Hence, the woman has difficulty touching her head and placing her hand behind her neck. This limitation seems to be caused by the pain of the traction exerted in the axillary cavity, scar, chest wall, and upper limb.

A study using computerized biophotogrammetry concluded that the most compromised range of motion in post-mastectomy women is shoulder flexion and that this disorder remained even six months after the operation. As a result of the muscle defense mechanism, there may be pain and muscle spasms in all the cervical region; the levator scapulae, teres major and teres minor, and infraspinatus muscles may present tenderness upon palpation, restricting active movement of the shoulder^(22)^.

A study that evaluated 148 patients submitted to axillary dissection along with the surgical procedure concluded that pain, loss of strength in the arm, and limitation of movements of the shoulder are frequent after axillary dissection, regardless of the type of operation the patient was submitted to. The study detected a difference of more than 20° in abduction, and ventral or dorsal elevation of the shoulder in 12% of the patients, and pain or loss of strength in half the patients^(8)^. In this study, shoulder pain was also reported, appearing in 52.5% of the patients of Group ML and in 41.8% of Group M. Additionally, there were reports of pain in the cervical, axillary, and thoracic spine regions. As seen, women with lymphedema have a higher frequency of pain when compared to those who did not develop the disease.

In a systematic review, Rietman et al.^(9)^ observed that the mobility of the shoulder was significantly less in patients who received radiation therapy in the axilla. Range of motion was significantly smaller in patients who underwent mastectomy when compared to those patients who were submitted to a more conservative treatment^(9)^.

In a comparative study between modified radical mastectomy (MRM) and breast conserving therapy (BCT) associated with radiation therapy, Nesvold et al.^(23)^demonstrated that 24% of the women submitted to MRM had restricted flexion of the shoulder, compared to 7% of those submitted to BCT. Shoulder pain was declared in 32% in MRM and in 12% in BCT. It was concluded that problems in the arm and shoulder, including lymphedema, are significantly more common after mastectomy.

## CONCLUSION

With these results, it was possible to conclude that women after mastectomy have alterations in posture and range of motion of the upper limb, especially in the shoulder. Lymphedema worsens such alterations, which was confirmed by comparing women who progressed with and without lymphedema.
